# Bee venom (*Apis mellifera* L.) rescues zinc oxide nanoparticles induced neurobehavioral and neurotoxic impact via controlling neurofilament and GAP-43 in rat brain

**DOI:** 10.1007/s11356-023-28538-1

**Published:** 2023-07-13

**Authors:** Naglaa Z. H. Eleiwa, Mahmoud Abo-Alkasem Ali, Enas N. Said, Mohamed M. M. Metwally, Yasmina M. Abd-ElHakim

**Affiliations:** 1 Department of Pharmacology, Faculty of Veterinary Medicine, Zagazig University, Zagazig, Egypt; 2 Department of Behaviour and Management of Animal, Poultry and Aquatic, Faculty of Veterinary Medicine, Zagazig University, Zagazig, 44519 Egypt; 3 Department of Pathology, Faculty of Veterinary Medicine, Zagazig University, Zagazig, 44511 Egypt; 4 Department of Forensic Medicine and Toxicology, Faculty of Veterinary Medicine, Zagazig University, Zagazig, Egypt

**Keywords:** Bee venom, Zinc oxide nanoparticles, Brain, Dopamine, Amyloid-β, Sensorimotor functions

## Abstract

This study investigated the possible beneficial role of the bee venom (BV, *Apis mellifera* L.) against zinc oxide nanoparticles (ZNPs)-induced neurobehavioral and neurotoxic impacts in rats. Fifty male Sprague Dawley rats were alienated into five groups. Three groups were intraperitoneally injected distilled water (C 28D group), ZNPs (100 mg/kg b.wt) (ZNPs group), or ZNPs (100 mg/kg.wt) and BV (1 mg/ kg.bwt) (ZNPs + BV group) for 28 days. One group was intraperitoneally injected with 1 mL of distilled water for 56 days (C 56D group). The last group was intraperitoneally injected with ZNPs for 28 days, then BV for another 28 days at the same earlier doses and duration (ZNPs/BV group). Depression, anxiety, locomotor activity, spatial learning, and memory were evaluated using the forced swimming test, elevated plus maze, open field test, and Morris water maze test, respectively. The brain contents of dopamine, serotonin, total antioxidant capacity (TAC), malondialdehyde (MDA), and Zn were estimated. The histopathological changes and immunoexpressions of neurofilament and GAP-43 protein in the brain tissues were followed. The results displayed that BV significantly decreased the ZNPs-induced depression, anxiety, memory impairment, and spatial learning disorders. Moreover, the ZNPs-induced increment in serotonin and dopamine levels and Zn content was significantly suppressed by BV. Besides, BV significantly restored the depleted TAC but minimized the augmented MDA brain content associated with ZNPs exposure. Likewise, the neurodegenerative changes induced by ZNPs were significantly abolished by BV. Also, the increased neurofilament and GAP-43 immunoexpression due to ZNPs exposure were alleviated with BV. Of note, BV achieved better results in the ZNPs + BV group than in the ZNPs/BV group. Conclusively, these results demonstrated that BV could be employed as a biologically effective therapy to mitigate the neurotoxic and neurobehavioral effects of ZNPs, particularly when used during ZNPs exposure.

## Introduction

Nanotechnology has become one of the most popular topics among scientists, producing many groundbreaking technologies (Sudhakaran et al. 2019). With the progress of nanotechnology, innovative novel nanoparticles (NPs) have been developed and have found success in a wide range of applications in different fields (Abd El-Hakim et al. 2020; Selim et al. 2015; Vance et al. 2015). Zinc oxide nanoparticles (ZNPs) are one of the most developed and useful nanomaterials in modern manufacturing processes (Dahran et al. 2023; Mahfouz et al. 2023).

ZNPs are widely utilized in disinfection, antibacterial, anti-ultraviolet, and deodorization products, medicine, electronics, gas sensors, and other industries thanks to their superior performance in light, magnetic, and electricity (Liu et al. 2020). Moreover, due to their potent antibacterial properties, ZNPs are often used in food packaging and even coated on dental implants (Shi et al. 2014). The extensive applications of ZNPs have raised concerns about their biosafety (Chen et al. 2020).

Prior research has established that ZNPs cause various harmful effects on mammalian systems. For instance, ZNPs induced genotoxic effects in the pancreatic and stomach cells when orally administrated daily at a dose of 422 mg/kg b.wt for 8 weeks in adult male albino rats (Abdallah 2018). Additionally, Tang et al. (2019) verified that oral administration of ≥ 50 mg/kg b.wt ZNPs (30 nm diameter) for 14 days in adult male mice caused spermatogenesis abnormalities, testicular damages, and reduced serum testosterone levels. Moreover, a significant increase in the serum levels of inflammatory cytokine (IFN-γ, IL-6, and TNF-α), the CD^4+^ and CD^8+^ cells number, and the Zn levels in the liver, spleen, and thymus of male BALB/c mice orally exposed to 50 mg ZNPs /kg b.wt for 14 consecutive days (Senapati et al. 2017).

Neurobehavioral disorders, including altered spatial learning and impaired memory were recorded in Sprague–Dawley rat pups on the 60^th^ postnatal day when their dams were orally exposed to 500 mg/kg b.wt ZNPs for 18 consecutive days on 2^nd^ –19^th^ gestational days (Xiaoli et al. 2017). Additionally, ZNPs caused major changes in the brain monoamines (serotonin, norepinephrine, and dopamine) and ions such as Zn^2+^, K^+^, Na^+^, and Ca^2+^ in male Swiss albino mice intraperitoneally injected 5.6 mg ZNPs /kg b.wt once daily for 5 days (Dkhil et al. 2020). Further, the in vitro study of Liu et al. (2020) on the PC12 cell line showed that 6 h exposure to ZNPs (2- 20 μg/mL) affected cell morphology, reduced cell viability, augmented oxidative stress activity levels, weakened mitochondrial function, and upset the cell cycle.

Therapeutic interventions have been sought for decades to stop the worsening of neurobehavioral disorders and brain injuries or speed their recovery (Abd-Elhakim et al. 2021, 2020; Behairy et al. 2020; Dahran et al. 2023). Toxins from venomous organisms such as spiders, snakes, scorpions, bees, toads, ants, wasps, and frogs have shown great promise for pharmaceutical uses (de Souza et al. 2018b). Bee venom (BV), one of the most beneficial biological toxins, has been demonstrated to have anticancer (Son et al. 2007) and antimutagenic (Hoshina and Marin-Morales 2014) properties. Moreover, BV has been studied as a possible therapy for treating numerous immune-related disorders in animals and, in the case of rheumatoid arthritis, in humans (Lee et al. 2014). Furthermore, BV exhibited antiviral, antibacterial, and antioxidant effects in various animal models (Alkhalefa et al. 2021; Hegazi et al. 2014; Mohamed et al. 2019). BV comprises multiple components with distinct biological roles and pharmacological effectiveness, like melittin, phospholipase A2, and apamin (Zidan et al. 2018).

Many clinical experiments have recently revealed that BV injection may be useful in the treatment of neurodegenerative disease models like amyotrophic lateral sclerosis (Mirshafiey 2007), Parkinson’s disease (Maurice et al. 2015), Alzheimer’s disease (Ye et al. 2016), and autism spectrum disorders (Daghestani et al. 2017). Furthermore, prior research has verified the beneficial role of BV in alleviating neurobehavioral disturbances connected to inflammation and pain (Merlo et al. 2011; Roh et al. 2006), preventing amphetamine addiction (Kwon et al. 2010), and neuroprotective properties against neurodegeneration (Kim et al. 2011).

Based on the pharmacological activities of BV, we hypothesized it could be an alleviative or curative intervention for the ZNPs-induced neurobehavioral disorders and neurotoxic impacts in rats. To test this hypothesis, several behavioral tests, biochemical indicators, and histopathological and immunohistochemical evaluations were performed on rats exposed to ZNPs and/ or BV.

## Material and methods

### Drugs and chemicals tested compounds

ZNPs (Sr. No. AL4235A00010, Batch No: ZP 322, molecular weight (MW) = 81.39; average nanosize 30 ± 5 nm particle size) were obtained from Alpha Chemika (Mumbai, India). Dried pure BV obtained from Lamarck’s honey bee or the Egyptian honey bee (*Apis mellifera lamarckii*) was purchased from the Bee Venom Pharma Company (El Obour City, Egypt). Based on the certificate of analysis of the manufacturer, BV comprised 1.639 mg/mL of protein, and high-performance liquid chromatography (HPLC) examination revealed the presence of melittin (68.12%), phospholipase A2 (10.54%), and apamin (1.52%). A stock solution of BV was prepared by dissolving in distilled water. All used reagents and chemicals were of analytical grade and obtained from Sigma–Aldrich Co. (St. Louis, Missouri, USA).

### ZNPs characterization and suspension preparation

The particle size and shape of the synthesized ZNP were explored using the scanning electron microscope (SEM, JSM-6701F Plus, JEOL) and transmission electron microscope (JEOL, TEM-2100, Japan) (Hassan et al. 2023). The ZNP dilute suspension was sonicated for about one h before the investigation to ensure the uniform dispersion of the particles. One or two drops of the sonicated suspension were dropped onto the grid and left for drying in the air before the examination.

The ZNPs particles were dispersed in distilled water (10 mg/mL). Following the protocol of Ramadan et al. (2022), a fresh suspension was prepared daily, and the suspensions were sonicated for 15 min with an ultrasonic cleaner (500 W, 42 kHz, 25 °C, FRQ-1010HT, Hangzhou, China). The prepared ZNPs suspension was stirred on a vortex agitator directly before animal administration. The earlier method has achieved high Zn dispersion stability (Thonglerth et al. 2022).

### Animals and experimental design

The animal studies detailed afterward were carried out following the National Institutes of Health’s general criteria for the care and use of laboratory animals in scientific investigations and were approved by the Ethics Committee of the Ethics of Animal Use in Research Committee (IACUC), Zagazig University, Egypt, with the reference number (ZU-IACUC/2/F/182/2021). Fifty Sprague Dawley male rats (12 weeks old and 148.2 ± 1.28 g) obtained from the Laboratory Animal Housing Unit, the Faculty of Veterinary Medicine, Zagazig University, Egypt were used. In a well-ventilated room, the rats were kept in stainless steel cages (45 cm × 28 cm × 18 cm) with a 12-h light/dark cycle, with free access to food and water. They were housed up to four per cage. The experimental animals were habituated to the laboratory setting for two weeks before being used in any of the procedures reported here.

The rats were distributed into five groups, each comprising 10 rats. The first group (C 28D) was intraperitoneally injected with 1 mL distilled water /rat for 28 days. The 2^nd^ group (C 56D) was intraperitoneally injected with 1 mL distilled water /rat for 56 days. The third group (ZNPs) was intraperitoneally injected ZNPs (100 mg/kg.wt) for 28 days. The 4^th^ group (ZNPs + BV) was intraperitoneally injected ZNPs (100 mg/kg.wt) and BV (1 mg/ kg.bwt) for 28 days. The 5^th^ group (ZNP/BV) was intraperitoneally injected with ZNPs for 28 days, then BV for another 28 days at earlier doses and duration. BV and ZNP injections were performed daily. The rats were weighed once a week, and their dosages were adjusted accordingly. Rats were observed for symptoms of pain, distress, abnormal behavior, irregular breathing, sickness, mucous membrane color, and death throughout the study.

### ZNPs and BV dose selection

Several earlier reports confirmed the ZNPs induced brain oxidative stress and neurobehavioral disturbances at the same tested dose (100 mg/kg b.wt). For instance, Rahdar et al. (2020) demonstrated that exposure to 100 mg ZNPs /kg for 28 days significantly increased brain MDA but significantly decreased brain catalase and superoxide dismutase activity. Moreover, the same authors confirmed that ZNPs caused prominent changes to brain histology and neurobehavioral defect including anxiety and locomotion. Additionally, Moshrefi et al. (2021) reported that the rats intraperitoneally injected 100 mg ZNPs /kg twice a week over 28 days showed increase zinc accumulation in their brain tissues together with reduced behavior index and increased oxidative stress. At the same time, it is well documented that under oxidative stress condition in the brain tissues, the reactive oxygen species (ROS) increase, the intracellular signaling is disturbed, and consequently the proinflammatory response is propagated which is the key to neurodegenerative diseases progression (Solleiro-Villavicencio and Rivas-Arancibia 2018; Teleanu et al. 2022).

Several previous reports established the antioxidant and anti-inflammatory activity of BV when intraperitoneally injected at the same tested dose (1 mg/kg b.wt). For example, Mohammed and Hassan (2019) reported that BV attenuated the inflammatory reactions when injected in rats at a dose of 1 mg/kg b.wt via the intraperitoneal route for 14 days. Besides, intraperitoneal injection of BV obtained from Egyptian honey bee (*Apis mellifera*) at the same tested dose for 6 successive weeks significantly counteracted diabetes- induced oxidative stress and inflammation (Hassan et al. 2019).

### Behavioral examination

All behavioral tests were conducted in a testing room adjacent to the laboratory room where rats reside. The testing room is acoustically insulated with controlled temperature (22–25 °C) and light (12 h light cycle). The investigator did not identify the treatment administered to each group. The behavioral tests were performed on two successive days. The open field test (OFT) and Morris water maze (MWM) were performed on the first day. Then, the elevated plus maze (EPM) and forced swimming test (FST) were carried out on the second day. On test days, rats were relocated from their cages to the testing room and allowed about 30 min to acclimate before the testing activities began. After each test, the rat was returned to its cage, and the apparatus was wiped with a moist sponge to remove odor.

#### Open field test

In line with de Souza et al. (2017) protocol, OFT has been used to assess anxiety. For this purpose, a 60 cm × 40 cm open-field arena containing a white floor alienated into 24 squares (15 cm × 15 cm) and surrounded by 40 cm-high walls was used. Each rat was placed in the center of the test arena, which the animal had never seen before. For 5 min, the number of central (away from the walls) and peripheral (close to the walls) squares touched by the rat’s four paws was counted. Central-quadrant time as a percentage of the total time was determined. Based on Prut and Belzung (2003), the time spent in the central quadrants and that spent in the lateral quadrants can be used to assess anxiety activity. The arena was thoroughly cleaned with a 10% ethanol solution after each test session.

#### Morris water maze

Morris water maze was used to assess memory and spatial learning on the last five days of the experiment (four training days and the last test day) in line with the protocols of Xie et al. (2012b) and da Silva et al. (2018). The MWM comprised a tank 60 cm in height and 120 cm in diameter, filled to a depth of 45 cm with water. To make the water opaque and to confirm that the scape platform would be hidden, nontoxic brown ink was added. Two imaginary perpendicular lines intersecting in the tank’s center created four equal quadrants labeled “I,” “II,” “III,” and “IV”. In the center of quadrant III, a black platform (5 cm in diameter) was placed in the target quadrant. The platform was 1–2 cm beneath the water’s surface. The MWM comprised two sections: place navigation and spatial probe. In the place navigation section: rats were trained to find the hidden platform. Each animal was "trained" to locate the hidden scape platform in the tank for four consecutive training days, with four attempts per training/day. In each training session, the rats were placed in the water with their noses turned to the tank’s edge. But the starting positions were switched around so that no rat ever started from the same spot twice in one day. Besides, the sequence would not be repeated in subsequent days. Rats were given 60 s to swim to the platform until they found it. If the rat did not locate the scape platform within this time frame, it would be led there by the researcher and given 15 s to explore the area. The animal was then taken off the platform and dried. The animals’ fecal pellets were cleaned out in between training sessions. After a 1-min break, it was returned to the tank for the next session. Each training day, the rat’s latency from entering the water to finding the scape platform was recorded. After all training sessions, the rats were dried and returned to their boxes every training day.

During the spatial probe on the test day, the scape platform was removed from the tank, rats were given another for 60 s to swim from the quadrant opposite to the target quadrant in the tank without the platform. The following parameters were determined: escape latency, latency to reach the target quadrant, target quadrant spent time, and the number of trials reaching the target quadrant. All training and testing took place during the light phase of the light–dark cycle. Furthermore, all test sessions were videotaped with a camera placed 100 cm above the tank.

#### Elevated plus maze

The EPM was used to measure anxiety in animals following the protocol followed in the study of de Souza et al. (2018a), with some modifications in the dimensions to match the rat size. The EPM device was made of wood, stood 50 cm above the floor, and consisted of two opposing open arms (50 × 10 cm) and two opposing closed arms of the same size. The arms were joined via a central arena (10 × 10 cm). The open arms were enclosed by a 0.5 cm high edge to keep the rat from falling. After that, each rat was put in the EPM device, facing one of the open arms. Animals were given 5 min to explore the equipment freely, and each rat was tested only once. Before each test, the EPM was disinfected with 10% ethanol. The total number of entries, defined as the number of times the animal entered the closed and opened arms, was used to account for the animals’ locomotor activity in this test. Besides, the anxiety index was determined using the following formula:

Anxiety index = 1 − [([time the animal remained in the open arms (seconds)/test duration (300 s)] + [input frequency in the open arms/total number of entries])/2].

#### Forced swimming test

The FST involved placing each rat separately in a cylindrical tank (40 cm height, 20 cm diameter) filled with water set at room temperature of 25 °C (Can et al. 2012; da Luz et al. 2022; da Silva et al. 2018) (20 cm depth) for 6 min. Then the rats were then taken out of the water, dried, and returned to their cages. A video camera placed 30 cm above the tank captured all sessions. This procedure allowed the recording of the time the rat remained immobile. The rat was described as immobile when it ceased struggling and floated on the water without making any noticeable movements other than those required to keep its head above the surface. Six water tanks were used. Each experimental group was tested in two sessions. Three rats were tested at one time using three tanks where dividers were placed between tanks to prevent rats from seeing each other in the test. The second water tank group was filled while the first set was in use. Then, the tank’s water was changed during the intermission between experimental groups. In FST, immobility is widely utilized as a depression predictor (Slattery and Cryan 2012).

### Sample collection and preparation

At the end of the experiment, the rats were anesthetized by intraperitoneal injections of 100 mg/kg b.wt pentobarbital sodium and euthanized by decapitation. The brain tissue samples were then dissected and washed in physiologic saline. Following saline washing, the brain tissue samples were divided into three sets. The first was homogenized, centrifuged at 664 × g at 4 °C for 15 min, and the resultant supernatant was used for biochemical analysis. The second was kept in 10% neutral buffered formalin for histopathological and immunohistochemical evaluations. The last one was preserved frozen till the quantification of the Zn amount.

### Biochemical assessment of brain tissue homogenate

#### Evaluation of oxidant/antioxidant status

Malondialdehyde (MDA) was evaluated in the homogenate brain tissue based on Ohkawa et al. (1979) protocol using Biodiagnostic(Dokki, Giza, Egypt) kits (CAT. No. MD 25 29). Thiobarbituric acid reacts with MDA in an acidic medium for 30 min at 95 °C to form thiobarbituric acid reactive product. The resulting pink product absorbance can be measured at 534 nm.

Total antioxidant capacity (TAC) was assessed using kit reagents (CAT. NO. TA 25 13, Biodiagnostic Co. Dokki, Giza, Egypt), as per Koracevic et al. (2001)’s methodology. To determine the sample’s antioxidative ability, the sample antioxidants are reacted with an exogenously provided, constant concentration of hydrogen peroxide (H_2_O_2_). A definite amount of the given H_2_O_2_ is neutralized by the antioxidants in the sample. An enzyme process involving the conversion of 3,5, dichloro -2- hydroxy benzensulphonate to a colorful product is used to colorimetrically measure the amount of H_2_O_2_ that is still present.

#### Assessment of brain dopamine and serotonin levels

In the brain tissue homogenate, dopamine has been evaluated using ELISA Kit (Biorbyt Ltd, Cambridge, United Kingdom) (Catalog no. orb410818, detection range: 0.156–0 ng/mL sensitivity: < 0.039 ng/mL). Also, serotonin brain content was estimated using rat serotonin (5HT) ELISA Kit (AssayGenie, Dublin, Ireland, Catalog no. RTEB1749, detection range: 0.78–50 ng/mL).

### Analysis of Zn residues

Pure certified metals, nitric acid (HNO_3_) 65% (Merck KGaA, Darmstadt, Germany), and perchloric acid (HClO_4_) (Merck KGaA, Darmstadt, Germany) were used to prepare standard solutions of varying concentrations, which were then used to plot the calibration curve. All plastic and glass containers were washed multiple times in distilled water, soaked in 10% HNO_3_, and finally rinsed in deionized water before use. The brain tissue samples were digested using the wet method (Choi et al. 2015). Briefly, 1 g of brain tissue with 20 mL of HNO_3_ and HClO_4_ mixture (4:1) was placed in a beaker and heated to 100 °C until the complete digestion of the sample. Then, to each digest, 10 mL of deionized water was added. The blank samples went through the same digestion procedures. The National Institute of Standards and Technology (NIST) standard reference material (NBS-bovine liver, No.1577 a) was employed to control the technique’s precision and accuracy. The recovery rate was 98% when the measured amounts of Zn were compared to those from the standard material. A Buck scientific model, 210 VGp flame with atomic absorption spectrophotometer, was used to read the absorbance and concentration digitally.

### Histopathological evaluation

The collected brain tissue specimens were fixed in 10% phosphate-buffered formalin for 48 h, thoroughly washed in distilled water, dehydrated in ethyl alcohol, cleared in HistoChoice® clearing agent (scientific laboratory supplies Ltd., England), impregnated and embedded in paraffin wax, sectioned at 5 µm thick, and stained with hematoxylin and eosin (Suvarna et al. 2018). For H&E-stained sections, five slides were made per animal using step serial sectioning (each section taken at 20 µm apart). The stained sections were examined by light microscope, and numerical multiparametric lesion scoring was carried out. To reduce bias, all slides intended for pathologic examination were sent to a pathologist without identification (blind examination). Concisely, the frequencies of the following lesions; neuronal chromatolysis, shrinkage, and necrosis, perineuronal vacuolation, neuropil vacuolation, astrogliosis, microgliosis, satellitosis, neuronophagia, vascular congestion, hemorrhage, endothelial hypertrophy, and inflammatory cell infiltration were quantified in five randomly selected non duplicated microscopic high-power fields (40 ×) per rat (fifty images per group). The results were presented as percentages (means ± SE).

### Neurofilament and GAP-43 immunohistochemical investigation

Two consecutive formalin-fixed paraffin-embedded 5 μm thick brain tissue sections were obtained for each rat. After heat-mediated antigen retrieval, one section was stained for neurofilament using the recombinant anti-neurofilament heavy polypeptide antibody [EP673Y] (ab40796) at 1/8000 dilution. The other was stained for growth- and plasticity-associated protein (GAP-43) and the recombinant anti-GAP-43 antibody [EP890Y] (ab75810) at 1:3000 dilution (Abcam Inc.) primary antibodies, respectively. For the immunohistochemically stained sections, two slides were obtained for each animal (one slide was stained for neurofilament, and the second slide was stained for GAP-43). The staining procedures were done according to the avidin–biotin-peroxidase complex immunohistochemical technique developed by (Hsu et al. 1981)**.** The reaction products were visualized through 3,3′-Diaminobenzidine (DAB) chromogen, and the nuclei were counterstained by hematoxylin stain. Next, the degree of neurofilament and GAP-43 immunoexpression in the cerebral cortices were quantified using the open-source Java image processing and analyzing software, ImageJ version 1.33, in five non-overlapped randomly selected microscopic fields for each animal (50 images per marker per group). Concisely, the images were captured at the same exposure time, same objective lens (40 ×), and the same camera (AmScope digital camera), and the images were analyzed by ImageJ calculating the percentages of the brown color positively stained neurofilament and GAP-43 areas fractions concerning the total areas of the images.

### Data analysis

The Kolmogorov–Smirnov and Levene’s tests were used to check the data for normality and variance homogeneity, respectively. When normality assumptions were met, using IBM SPSS Statistics, version 21 (IBM; Armonk, New York, USA)(Spss 2011), data were analyzed by one-way analysis of variance (ANOVA) to statistically define the variation between groups, followed by Tukey’s multiple range post hoc test for pairwise comparisons. The data has been displayed as means ± SE for each group. At *P* < 0.05, mean differences were considered significant. Moreover, GraphPad Prism version 8 (GraphPad Software, San Diego, CA, USA) was used for data presentation(Prism 2019). The principal component analysis was applied to the replicates of all analyses by the Granato et al. (2018) method. All the data of oxidative stress (TAC and MDA), neurotransmitters (serotonin and dopamine) GAP-43 and neurofilament immunoexpression level, and brain Zn content and the representative neurobehavioral indicators of anxiety (latency in outer edge in OFT and anxiety index in EPM), depression (immobility time in FST), and memory (number of trials in MWM) were included in the PCA analysis. The data were normally distributed and thus were not transformed.

## Results

### Characterization of ZNP

**Fig. 1:**
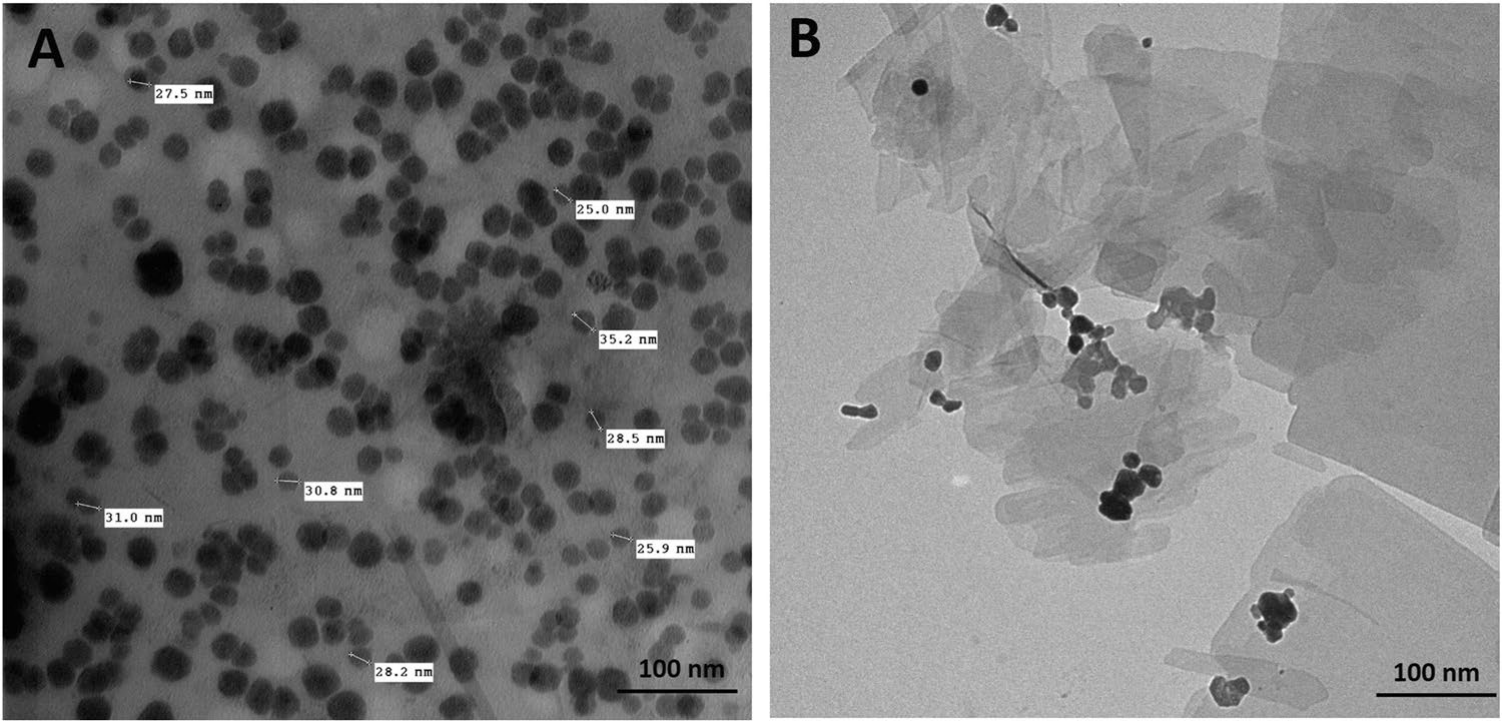
(**A**) The scanning electron microscope (SEM) and (**B**) transmission electron microscope (TEM) photos for the shape and size of zinc oxide nanoparticles

The particle size and shape of ZNPs were evaluated using SEM and TEM measurements, and the results were indicated in Fig. 1. The ZNPs were spherical in geometry within the range of 25–35 nm with an average diameter of 30 nm.

### Changes in body weight gain and brain index

As presented in Table 1, compared to the C28D control group, ZNPs-administered rats showed a significant reduction (*P* = 0.004) in daily weight gain. On the other hand, ZNPs + BV-treated rats showed a significant increment in daily weight gain compared to ZNPs exposed rats. Moreover, no significant difference exists in daily weight gain between ZNPs + BV and ZNPs/BV-treated group and the C28D and C56D control groups, respectively. On the other hand, no significant in brain weight was recorded among different experimental groups.

**Table 1:** Effect of bee venom (BV) and/or zinc oxide nanoparticles (ZNPs) intraperitoneal (IP) treatments on the body weight change and brain weight

Lesion	C 28D	C 56D	ZNPs	ZNP + BV	ZNP/BV
Initial body weight (g)	144.88 ± 2.86	152.25 ± 1.86	148.50 ± 3.69	149.50 ± 3.64	145.88 ± 1.58
Final body weight (g)	178.25 ± 3.45	224.00 ± 7.61	170.50 ± 4.65	192.38^#^ ± 6.70	206.75^#$^ ± 3.03
Daily weight gain	1.19 ± 0.09	1.28 ± 0.15	0.79* ± 0.10	1.53^#^ ± 0.19	1.09 ± 0.07
Absolute brain weight (g)	1.51 ± 0.03	1.79 ± 0.02	1.48 ± 0.03	1.53 ± 0.02	1.69^#$^ ± 0.04
Relative brain weight	0.85 ± 0.00	0.81 ± 0.03	0.87 ± 0.02	0.81 ± 0.04	0.82 ± 0.02

Daily weight change = (Final body weight—Initial body weight)/ days of the experiment. C 28D group: IP injected distilled water for 28 days. C 56D group: IP injected distilled water for 56 days. ZNPs group: IP injected 100 mg ZNPs /kg b.wt for 28 days. ZNP + BV group: IP injected 100 mg ZNPs /kg b.wt and 1 mg BV/ kg.bwt for 28 days. ZNP/BV group: IP injected 100 mg ZNPs /kg b.wt for 28 days then with 1 mg BV/ kg.bwt for 28 days. Values are represented as the mean ± SE, *n* = 10 for each group. *P < 0.05 vs control (C 28D).^#^ *P* < 0.05 vs ZNPs. $ *P* < 0.05 vs control (C 56D)

### Effects on neurobehavioral performance

#### Anxiety

**Fig. 2:**
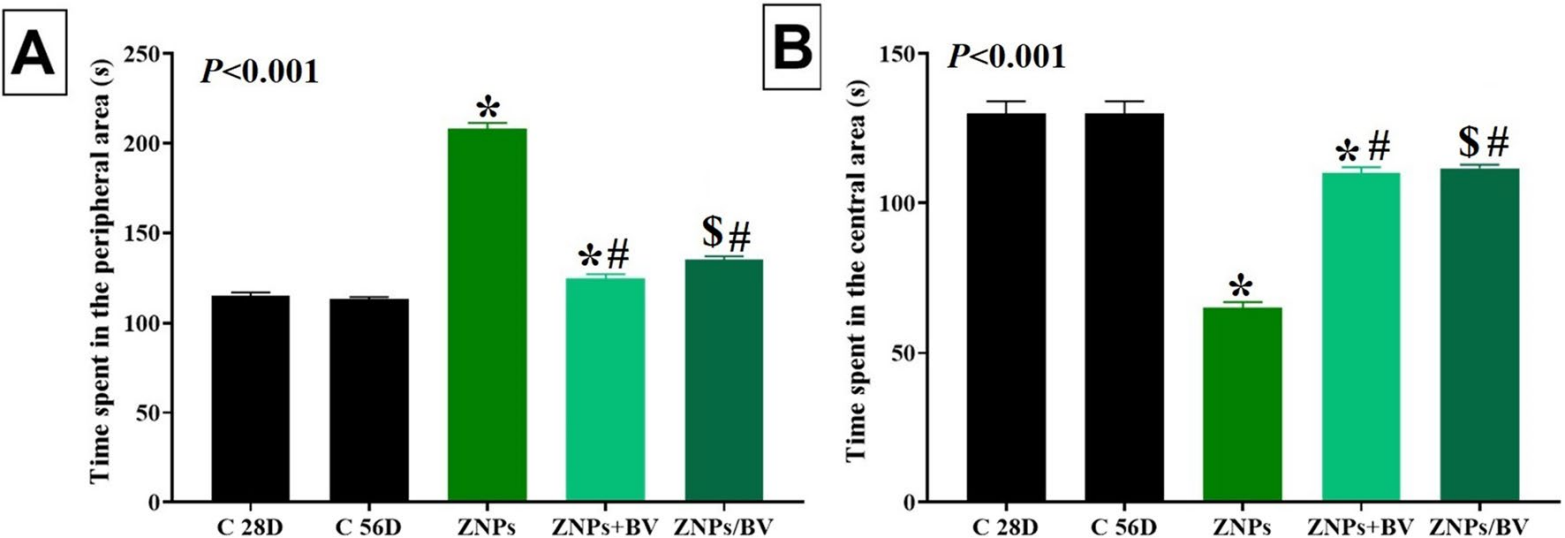
Effect of bee venom (BV) and/or zinc oxide nanoparticles (ZNPs) intraperitoneal (IP) treatments on time spent in the peripheral area (**A**) and the time spent in the central area (**B**) in the open field test. C 28D group: IP injected distilled water for 28 days. C 56D group: IP injected distilled water for 56 days. ZNPs group: IP injected 100 mg ZNPs /kg b.wt for 28 days. ZNP + BV group: IP injected 100 mg ZNPs /kg b.wt and 1 mg BV/ kg.bwt for 28 days. ZNP/BV group: IP injected 100 mg ZNPs /kg b.wt for 28 days then with 1 mg BV/ kg.bwt for 28 days. Values are represented as the mean ± SE, *n* = 6 for each group. *P < 0.05 vs control (C 28D).^#^ *P* < 0.05 vs ZNPs. $ *P* < 0.05 vs control (C 56D)

**Fig. 3:**
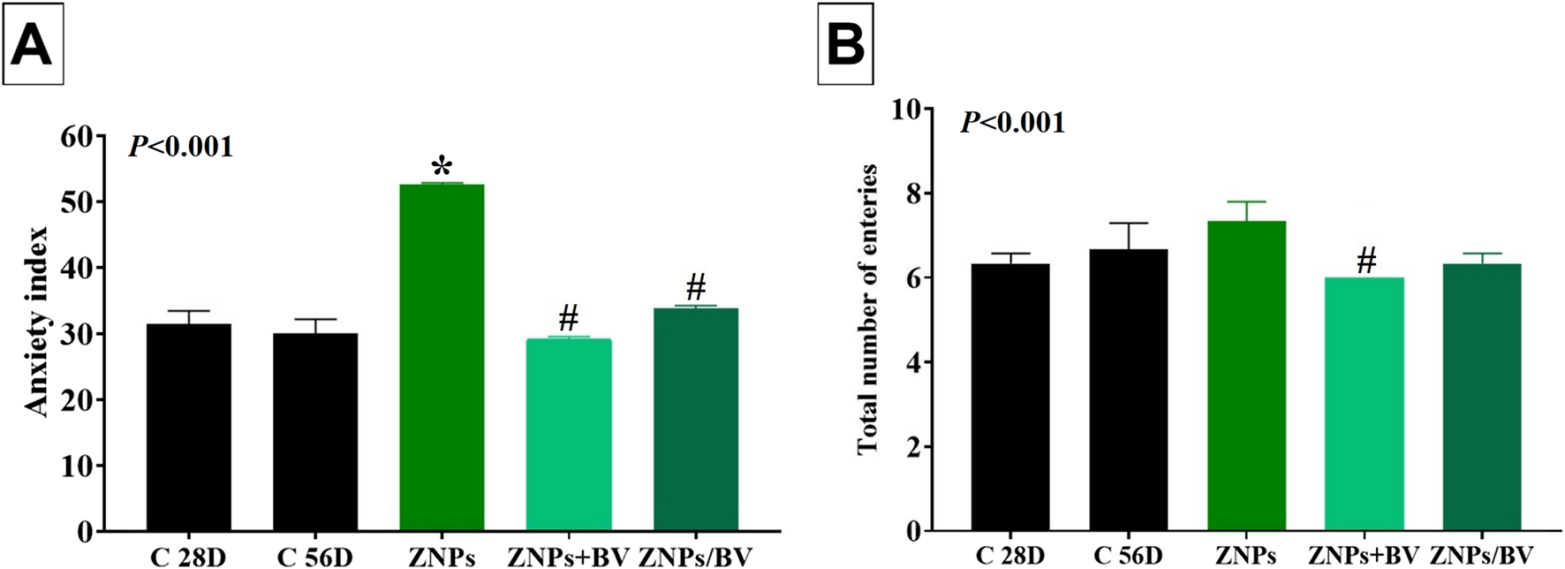
Effect of bee venom (BV) and/or zinc oxide nanoparticles (ZNPs) intraperitoneal (IP) treatments on the anxiety index (**A**) and the total number of entries (**B**) in the elevated plus maze test. C 28D group: IP injected distilled water for 28 days. C 56D group: IP injected distilled water for 56 days. ZNPs group: IP injected 100 mg ZNPs /kg b.wt for 28 days. ZNP + BV group: IP injected 100 mg ZNPs /kg b.wt and 1 mg BV/ kg.bwt for 28 days. ZNP/BV group: IP injected 100 mg ZNPs /kg b.wt for 28 days then with 1 mg BV/ kg.bwt for 28 days. Values are represented as the mean ± SE, *n* = 6 for each group. *P < 0.05 vs control (C 28D).^#^ *P* < 0.05 vs ZNPs. $ *P* < 0.05 vs control (C 56D)

The effect of BV injection as an alleviative or curative agent on ZNPs-induced anxiety was presented in the findings of OFT and EPM tests in Fig. 2 and 3, respectively. Initially, the ZNPs-exposed group showed a prominent anxiogenic effect as revealed by a significant (*P* < 0.001) increase in the time the rats spent in the peripheral area by 81.16% but reduced the time spent in the center area to 40.91% compared to the control group. Moreover, the anxiety index in the EPM was significantly (*P* < 0.001) increased by 67.04% in the ZNPs-exposed group compared to the control group. Nevertheless, the ZNPs + BV or ZNPs/BV treated rats displayed a significant (*P* < 0.001) reduction in the time spent in the peripheral area but increased the time spent in the center area compared to the ZNPs group. Moreover, the anxiety index was significantly (*P* < 0.001) reduced in the ZNPs + BV or ZNPs/BV treated group compared to the control group (Fig. 2A). Of note, the anxiety index was decreased in BV- injected groups to a level that no significant difference exists compared to the control group.

#### Spatial learning and memory

**Fig. 4:**
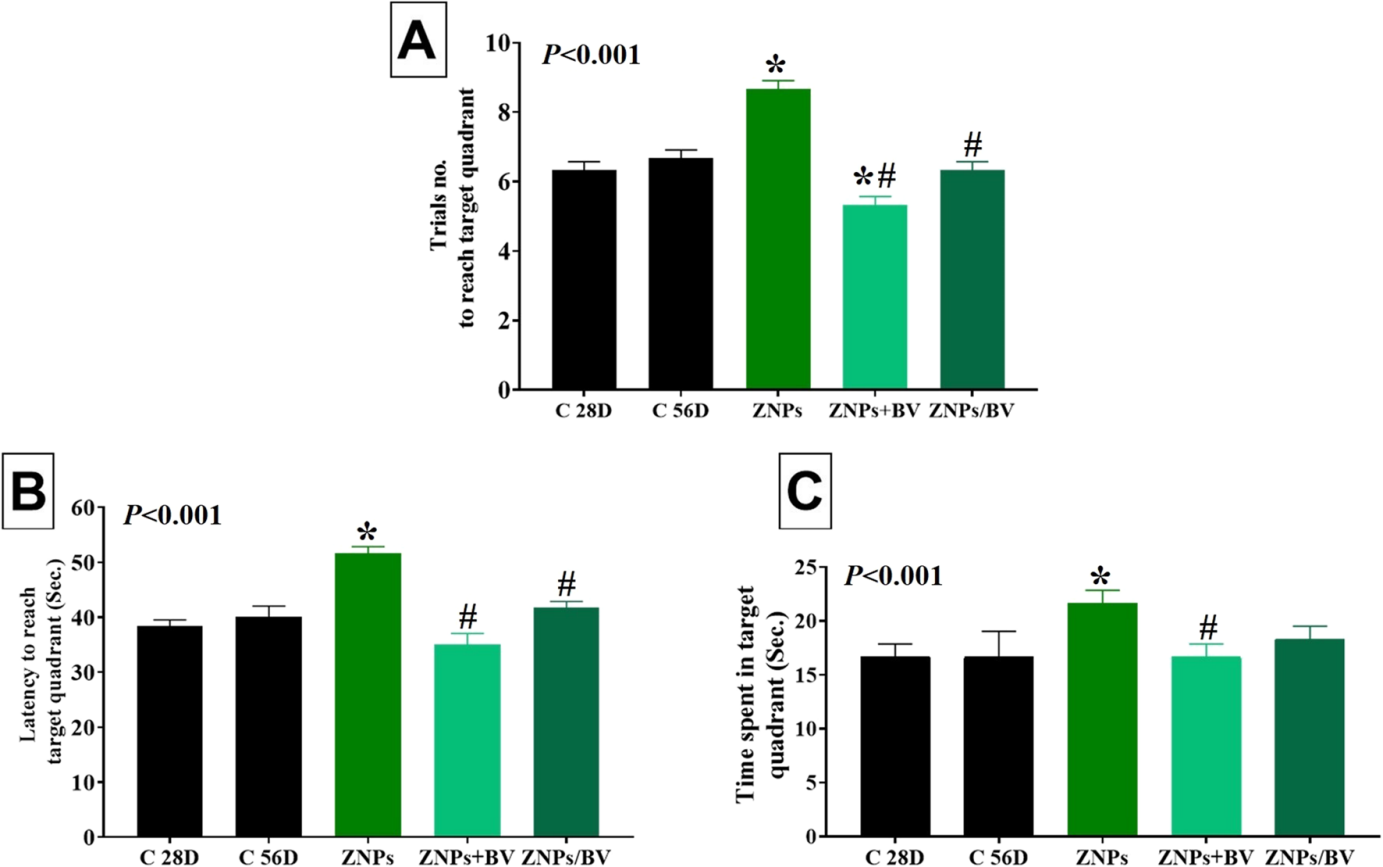
Effect of bee venom (BV) and/or zinc oxide nanoparticles (ZNPs) intraperitoneal (IP) treatments on the number of trials (**A**) and latency (**B**) to reach the target quadrant and the time spent in the quadrant (**C**) in the Morris water maze. C 28D group: IP injected distilled water for 28 days. C 56D group: IP injected distilled water for 56 days. ZNPs group: IP injected 100 mg ZNPs /kg b.wt for 28 days. ZNP + BV group: IP injected 100 mg ZNPs /kg b.wt and 1 mg BV/ kg.bwt for 28 days. ZNP/BV group: IP injected 100 mg ZNPs /kg b.wt for 28 days then with 1 mg BV/ kg.bwt for 28 days. Values are represented as the mean ± SE, *n* = 6 for each group. *P < 0.05 vs control (C 28D).^#^ *P* < 0.05 vs ZNPs. $ *P* < 0.05 vs control (C 56D)

The results of MWM, which indicate short-term memory and spatial learning, are shown in Fig. 4. The ZNPs-exposed rats had significantly (*P* < 0.001, 36.93%) more trials to reach the target quadrant and spent significantly (*P* < 0.001) more time to reach or stay in the target quadrant (34. 80% and 29.98%, respectively) compared to the control group. On the contrary, the ZNPs + BV-treated rats displayed a significant (*P* < 0.001) reduction in the trial number to reach the target quadrant and the time spent to reach or stay in the target quadrant compared to the ZNPs-exposed one. Additionally, a significant (*P* < 0.001) reduced trials number to reach the target quadrant and the time spent to reach the target quadrant was recorded in the ZNP/BV treated group compared to the ZNPs-exposed one.

#### Locomotor activity

The total number of entries in both closed and opened arms in EPM was used here to indicate rat locomotor activity. As demonstrated in Fig. 3B, a trend to increase in locomotor activity was recorded in ZNPs-exposed rats but was non-significant compared to the control group. However, the ZNPs + BV-treated rats showed significantly lower locomotor activity compared to ZNPs. Nonetheless, no significant difference was recorded between ZNPs and ZNP/BV in their locomotors activity.

#### Depression

**Fig. 5:**
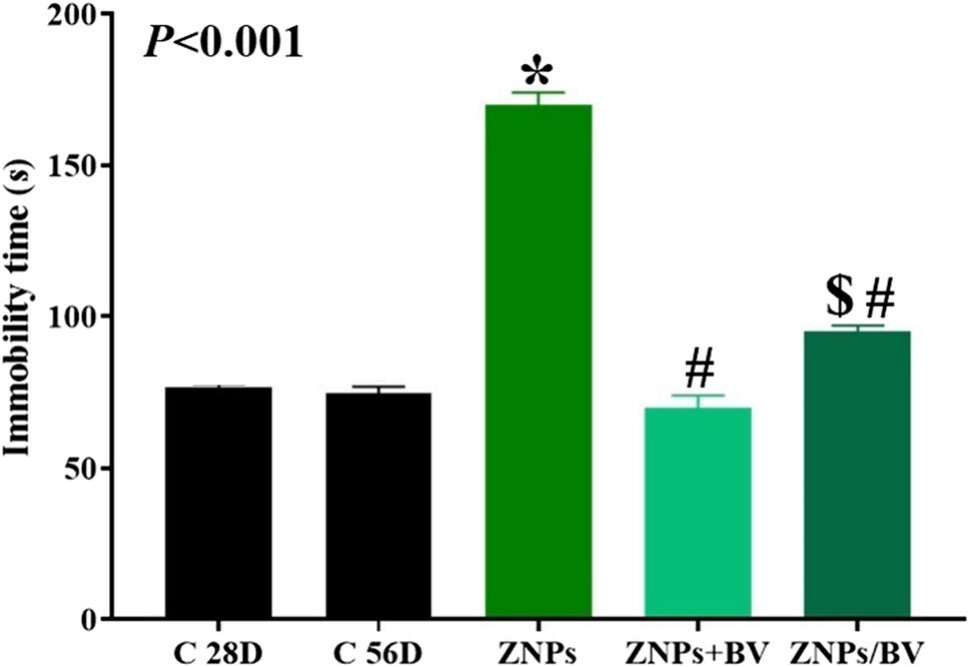
Effect of bee venom (BV) and/or zinc oxide nanoparticles (ZNPs) intraperitoneal (IP) treatments on immobility time in the forced swimming test. C 28D group: IP injected distilled water for 28 days. C 56D group: IP injected distilled water for 56 days. ZNPs group: IP injected 100 mg ZNPs /kg b.wt for 28 days. ZNP + BV group: IP injected 100 mg ZNPs /kg b.wt and 1 mg BV/ kg.bwt for 28 days. ZNP/BV group: IP injected 100 mg ZNPs /kg b.wt for 28 days then with 1 mg BV/ kg.bwt for 28 days. Values are represented as the mean ± SE, *n* = 6 for each group. *P < 0.05 vs control (C 28D).^#^ *P* < 0.05 vs ZNPs. $ *P* < 0.05 vs control (C 56D)

As demonstrated in Fig. 5, in the forced swim test, the ZNPs-exposed rats showed a significant increase in the immobility time by 121.73% compared to the control one. On the contrary, immobility reduction was detected in rats treated with BV to be 8.7% and 23.91% higher in the ZNPs + BV and ZNPs/BV-treated rats, respectively than the control group a significantly (*P* < 0.001) lower than the ZNPs-exposed rats.

### Changes in brain neurotransmitters

**Fig. 6:**
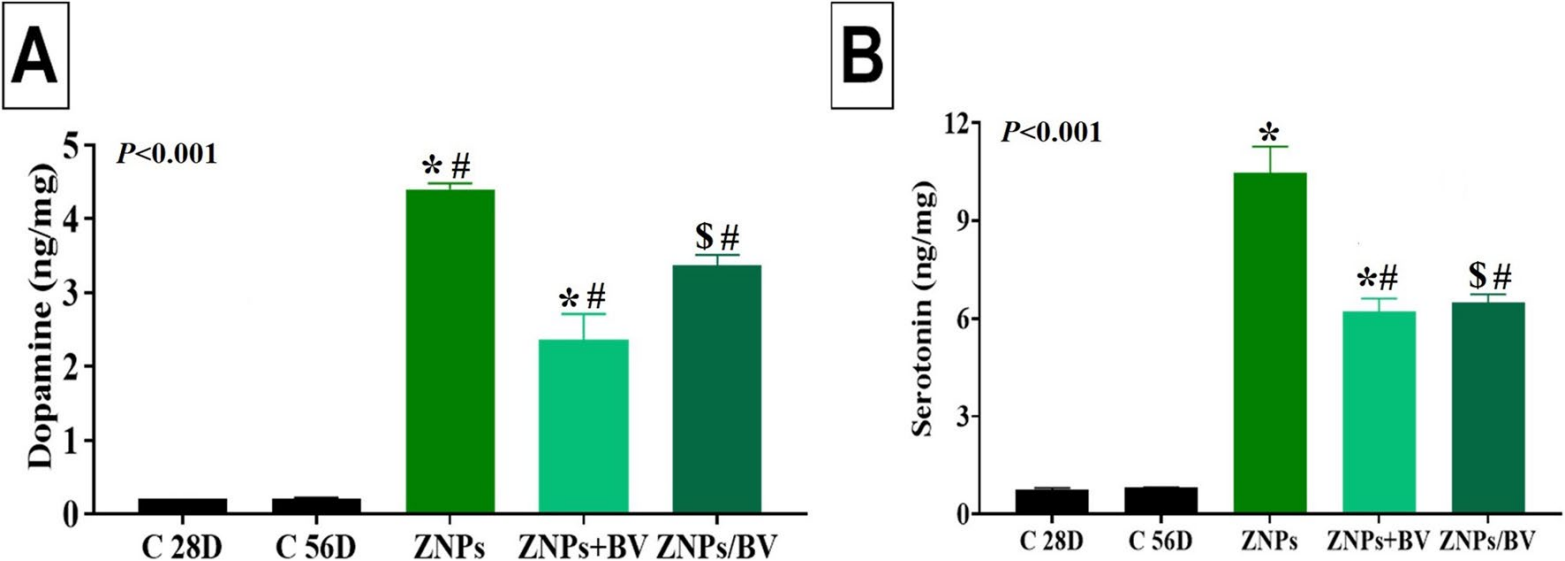
Effect of bee venom (BV) and/or zinc oxide nanoparticles (ZNPs) intraperitoneal (IP) treatments on brain tissue content of dopamine (**A**) and serotonin (**B**). C 28D group: IP injected distilled water for 28 days. C 56D group: IP injected distilled water for 56 days. ZNPs group: IP injected 100 mg ZNPs /kg b.wt for 28 days. ZNP + BV group: IP injected 100 mg ZNPs /kg b.wt and 1 mg BV/ kg.bwt for 28 days. ZNP/BV group: IP injected 100 mg ZNPs /kg b.wt for 28 days then with 1 mg BV/ kg.bwt for 28 days. Values are represented as the mean ± SE, *n* = 6 for each group. *P < 0.05 vs control (C 28D).^#^ *P* < 0.05 vs ZNPs. $ *P* < 0.05 vs control (C 56D)

The changes in the neurotransmitters (dopamine and serotonin) brain content in rats exposed to ZNPs and those treated with BV as an alleviative or curative agent were demonstrated in Fig. 6. The ZNPs-exposed rats had a significantly higher content of dopamine and serotonin by about 20 and 13 fold of the control rat’s values. In contrast, BV treatment significantly suppressed the ZNPs-induced increment in brain dopamine and serotonin levels to be 10, and sixfold of the control values in the ZNPs + BV and ZNPs/BV treated group, respectively, and a significantly (*P* < 0.001) lower than the ZNPs-exposed rats.

### Effects on brain oxidative stress and lipid peroxidation

**Fig. 7:**
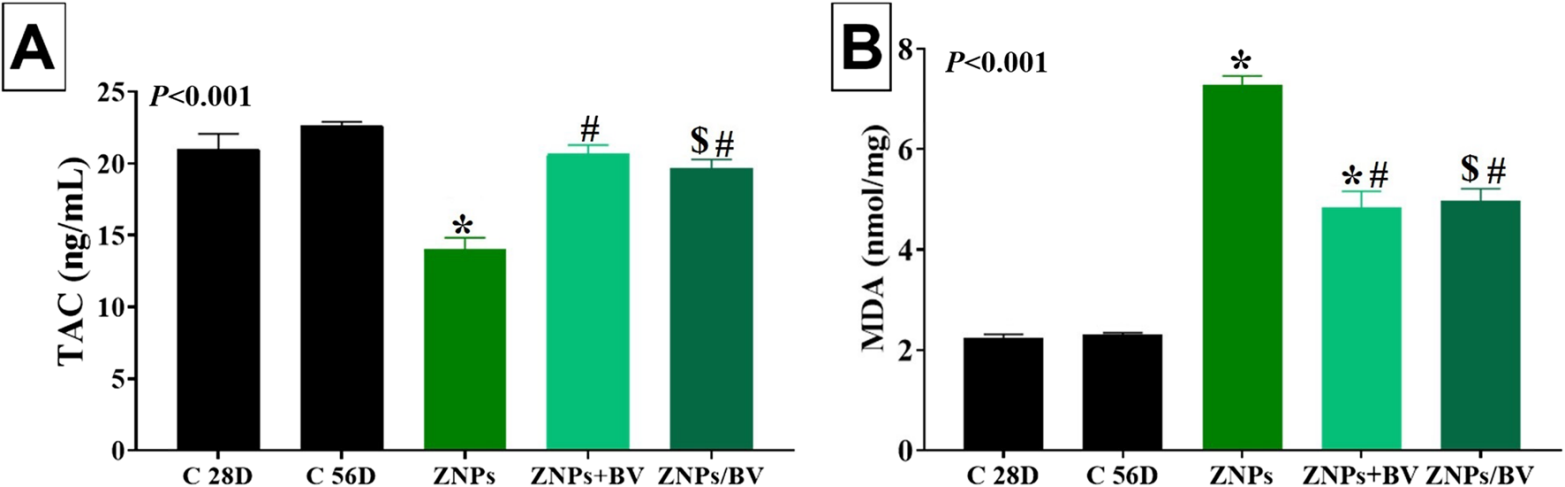
Effect of bee venom (BV) and/or zinc oxide nanoparticles (ZNPs) intraperitoneal (IP) treatments on brain tissue content of total antioxidant capacity (TAC) (**A**) and malondialdehyde (MDA) (**B**). C 28D group: IP injected distilled water for 28 days. C 56D group: IP injected distilled water for 56 days. ZNPs group: IP injected 100 mg ZNPs /kg b.wt for 28 days. ZNP + BV group: IP injected 100 mg ZNPs /kg b.wt and 1 mg BV/ kg.bwt for 28 days. ZNP/BV group: IP injected 100 mg ZNPs /kg b.wt for 28 days then with 1 mg BV/ kg.bwt for 28 days. Values are represented as the mean ± SE, *n* = 6 for each group. *P < 0.05 vs control (C 28D).^#^ *P* < 0.05 vs ZNPs. $ *P* < 0.05 vs control (C 56D)

The effect of BV administration as an alleviative or curative agent on the brain content of the TAC and the lipid peroxidation indicator (MDA) in rats exposed to ZNPs for 28 days was displayed in Fig. 7. The ZNPs-exposed rats exhibited a significant (*P* < 0.001) lower TAC level (33.33%) but higher MDA content (225.9%) relative to the control group. Nevertheless, the TAC was restored to a reduced level of 1.58%, and 6.35% in the ZNPs + BV and ZNPs/BV treated group, respectively, compared to the control group and a significantly (*P* < 0.001) higher than the ZNPs-exposed rats. Moreover, the TAC brain content was restored in the ZNPs + BV treated group to a level that no significant difference exists compared to the control group. Besides, BV significantly reduced the ZNPs-induced increase in the MDA content in the ZNPs + BV, and ZNPs/BV treated groups to 116.70% and 122.76%, respectively, compared to the control group and a significantly (*P* < 0.001) lower than the ZNPs-exposed rats.

### Changes in brain zinc content

**Fig. 8:**
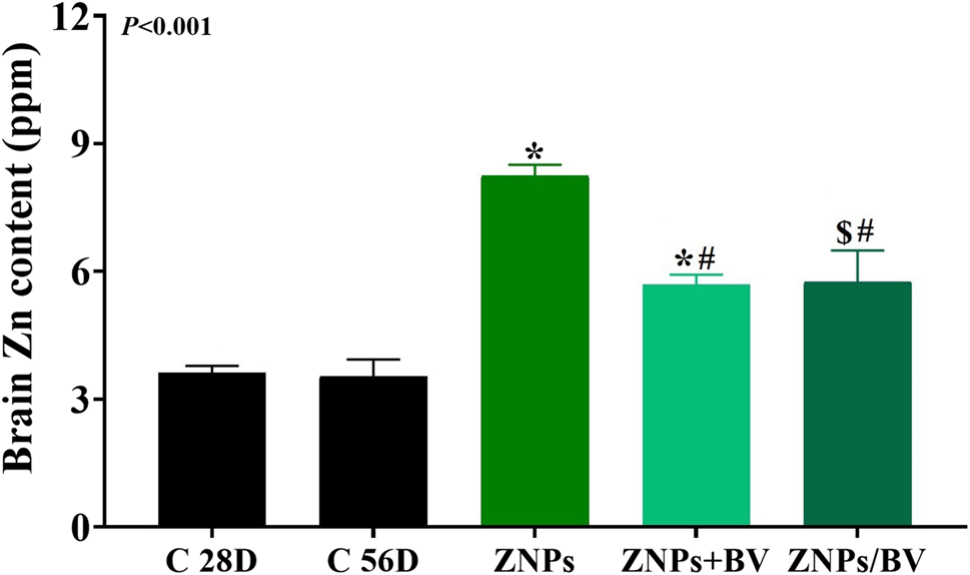
Effect of bee venom (BV) and/or zinc oxide nanoparticles (ZNPs) intraperitoneal (IP) treatments on brain tissue content of zinc (Zn). C 28D group: IP injected distilled water for 28 days. C 56D group: IP injected distilled water for 56 days. ZNPs group: IP injected 100 mg ZNPs /kg b.wt for 28 days. ZNP + BV group: IP injected 100 mg ZNPs /kg b.wt and 1 mg BV/ kg.bwt for 28 days. ZNP/BV group: IP injected 100 mg ZNPs /kg b.wt for 28 days then with 1 mg BV/ kg.bwt for 28 days. Values are represented as the mean ± SE, *n* = 10 for each group. *P < 0.05 vs control (C 28D).^#^ *P* < 0.05 vs ZNPs. $ *P* < 0.05 vs control (C 56D)

The alterations in the brain Zn content in rats exposed to ZNPs and those treated with BV as an alleviative or curative agent were revealed in Fig. 8. The ZNPs-exposed rats displayed a significant increase of Zn by 127.20% than the control group. On the contrary, BV significantly reduced the ZNPs-induced rise in the brain Zn content in the ZNPs + BV, and ZNPs/BV treated groups to 56.82% and 56.61%, respectively compared to the control group and a significantly (*P* < 0.001) lower than the ZNPs-exposed rats.

### Pathological findings

**Fig. 9:**
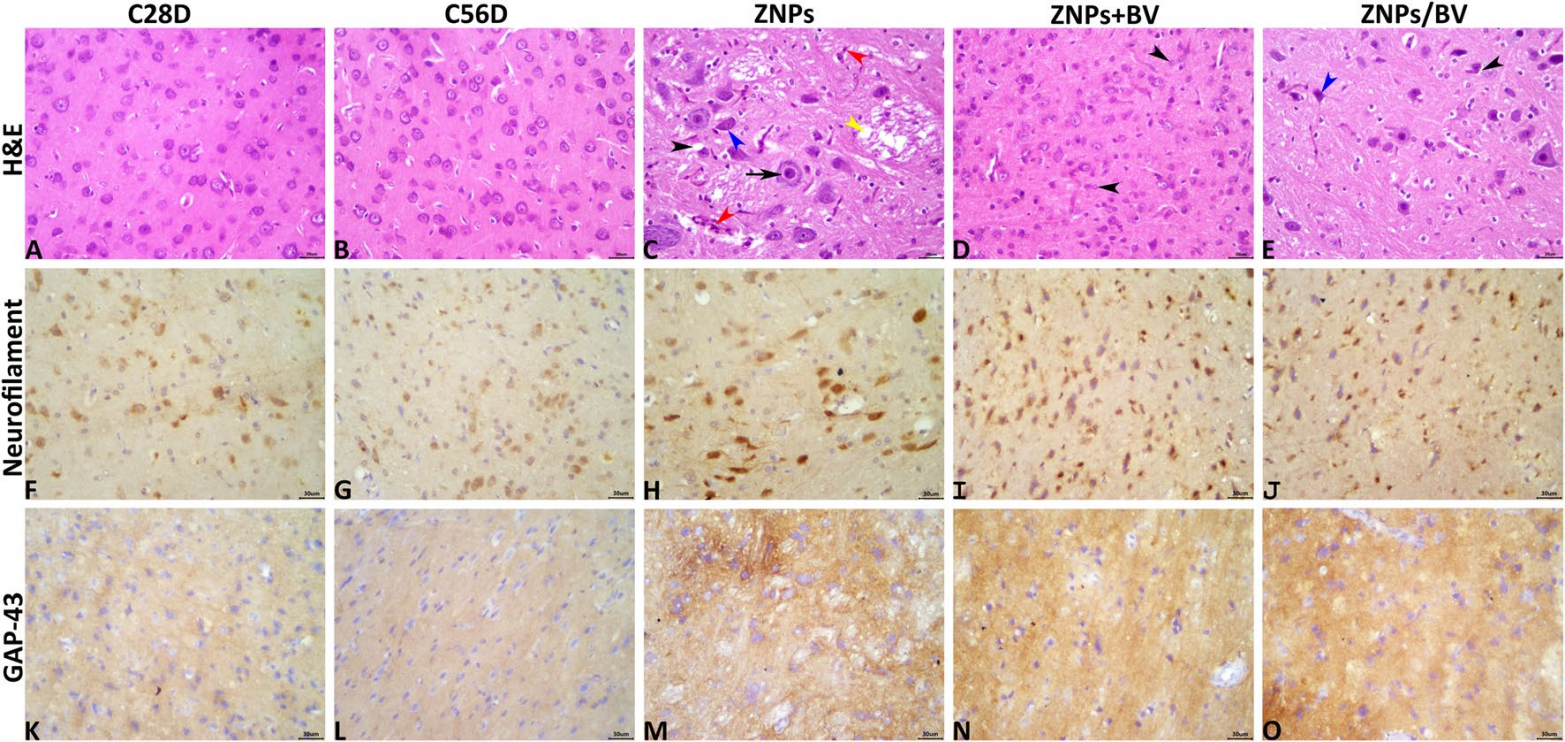
**A-E.** Representative photomicrograph of H&E-stained brain tissue sections in different experimental groups. C 28D group: IP injected distilled water for 28 days. C 56D group: IP injected distilled water for 56 days. ZNPs group: IP injected 100 mg ZNPs /kg b.wt for 28 days. ZNP + BV group: IP injected 100 mg ZNPs /kg b.wt and 1 mg BV/ kg.bwt for 28 days. ZNP/BV group: IP injected 100 mg ZNPs /kg b.wt for 28 days then with 1 mg BV/ kg.bwt for 28 days. The C 28D and C 56D control groups showing normal histological pictures in the cerebrums (**A**, and **B**). The ZNPs-treated group shows hypertrophied endothelium (red arrowheads), central chromatolysis (black arrow), perineuronal vacuolation (black arrowhead), neuropil microcavitation (yellow arrowhead), angular hypereosinophilic neuron (blue arrowhead) (**C**). The ZNP + BV treated group shows a nearly normal histological picture except for increased glial cells, particularly astrocytes (black arrowheads) (**D**). The ZNP/BV-treated group shows angular hypereosinophilic neuron (blue arrowhead0, and perineuronal vacuolation (black arrowhead) (**E**). Scale bars 30 microns. **F-J.** Representative photomicrograph of neurofilament-stained brain tissue sections showing upregulation of the neurofilament immunoexpression in the ZNPs (**H**), ZNP + BV (**I**), and ZNP + BV (**J**) groups compared to the C1 (**F**), and C2 (**G**) groups. Scale bars 30 microns. **K–O**. Representative photomicrograph of GAP-43-stained brain tissue sections showing upregulation of the GAP-43 immunoexpression in the ZNPs (**M**), ZNP + BV (**N**), and ZNP + BV (**O**) groups compared to the C1 (**K**), and C2 (**L**) groups. Scale bars 30 microns

Normal histological pictures were seen in the brains of the C28D and C56D control groups (Fig. 9A, and B). The brain tissue sections of the animals exposed to ZNPs (ZNPs group) showed numerous encephalopathic changes. Most of these changes were degenerative. The most frequent recorded lesions in this group were central chromatolysis, perineuronal vacuolation, neuropil microcavitation, gliosis, a few angular hypereosinophilic neurons, and hypertrophied endothelium (Fig. 9C). The brains of animals exposed to concurrent treatment with ZNPs, and BV (ZNP + BV group) showed a nearly complete absence of the ZNPs-induced encephalopathic alterations. Yet, the brains did not maintain their normal histology, as numerous sections showed an increase in the numbers of glia cells, particularly astrocytes (Fig. 9D). The brains of animals treated with BV after exposure to ZNPs (ZNP/BV group) showed nearly the same lesions seen in the ZNPs group. The only differences were some lesions’ reduced frequencies and severities, particularly the neuropil microcavitation (Fig. 9E).

### Immunohistochemical findings

The neurofilament and GAP-43 immunohistochemical positively brown stained surface area fractions in all experimental groups were presented in Fig. 9F–J, and K–O were quantified in Table 2. Statistically, exposure to ZNPs significantly upregulated the neurofilament and GAP-43 immunoexpression compared with the control (C28D, and C56D) animals. The immunoexpression of neurofilament and GAP-43 in the brains of animals treated with BV (ZNP + BV and ZNP/BV groups) showed slightly lower expression compared to the ZNPs group.

**Table 2:** Effect of bee venom (BV) and/or zinc oxide nanoparticles (ZNPs) intraperitoneal (IP) treatments on the brain histology, neurofilament, and GAP43 immunopositivity

Lesion	C 28D	C 56D	ZNPs	ZNP + BV	ZNP/BV
Neuronal chromatolysis	0.00 ± 0.00	0.00 ± 0.00	6.00* ± 2.56	2.00* ± 1.33	4.00^$^ ± 2.21
Neuronal shrinkage	0.00 ± 0.00	0.00 ± 0.00	8.00* ± 2.02	2.00 ^#^ ± 2.00	4.00 ± 2.21
Neuronal necrosis	0.00 ± 0.00	0.00 ± 0.00	2.00 ± 2.00	0.00 ± 0.00	0.00 ± 0.00
Perineural vacuolation	0.00 ± 0.00	0.00 ± 0.00	14.00* ± 4.27	2.00 ^#^ ± 2.00	4.00^#^ ± 2.67
Neuropil microcavitation	0.00 ± 0.00	0.00 ± 0.00	16.00* ± 5.81	4.00 ^#^ ± 2.67	6.00^#^ ± 3.06
Astrogliosis	0.00 ± 0.00	0.00 ± 0.00	6.00* ± 1.93	4.00 ± 2.21	4.00 ± 2.21
Microgliosis	0.00 ± 0.00	0.00 ± 0.00	2.00 ± 2.00	0.00 ± 0.00	0.00 ± 0.00
Hypertrophied endothelium	0.00 ± 0.00	0.00 ± 0.00	14.00* ± 3.55	6.00^#^ ± 3.06	6.00 ^#^ ± 3.06
Vascular congestion	0.00 ± 0.00	0.00 ± 0.00	6.00* ± 2.45	2.00 ± 1.11	4.00 ± 2.67
Hemorrhages	0.00 ± 0.00	0.00 ± 0.00	2.00* ± 0.73	0.00^#^ ± 0.00	0.00^#^ ± 0.00
Neurofilament area fraction	5.57 ± 0.27	6.53 ± 0.28	15.08* ± 0.29	11.28*^#^ ± 0.42	13.19^$#^ ± 0.32
GAP43 area fraction	3.53 ± 0.24	3.40 ± 0.28	20.35* ± 0.26	12.98*^#^ ± 1.93	16.19^$#^ ± 0.55

C 28D group: IP injected distilled water for 28 days. C 56D group: IP injected distilled water for 56 days. ZNPs group: IP injected 100 mg ZNPs /kg b.wt for 28 days. ZNP + BV group: IP injected 100 mg ZNPs /kg b.wt and 1 mg BV/ kg.bwt for 28 days. ZNP/BV group: IP injected 100 mg ZNPs /kg b.wt for 28 days then with 1 mg BV/ kg.bwt for 28 days. Values are represented as the mean ± SE, *n* = 10 for each group. *P < 0.05 vs control (C 28D).^#^ *P* < 0.05 vs ZNPs. ^$^*P* < 0.05 vs control (C 56D)

### Correlation analysis of the estimated parameters

**Fig. 10:**
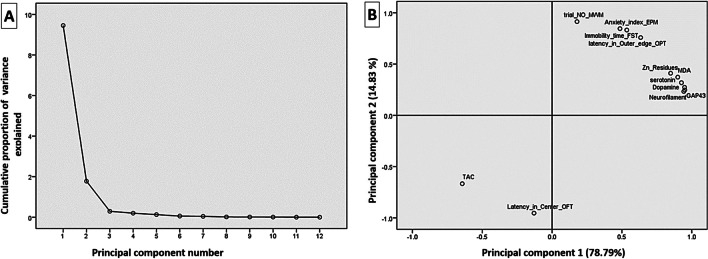
The principal component analysis plot shows the estimated variables’ relationships. (**A**) Cumulative proportion of variance as a function of the number of principal components (PC). (**B**) All biochemical and neurobehavioral indicators are plotted as a function of PC1 and PC2, which account for 78.79% and 14.83% of the variance, respectively. DOPA: dopamine; MDA: malondialdehyde; total antioxidant capacity (TAC); EPM: elevated plus maze test; FST: forced swimming test; OFT: open field test; Zn:zinc

Principal components analysis was used to examine the correlation between the analyzed variables. As shown in Fig. 10, the loading plot of the first two components (PC1 and PC2) represented approximately 93.62% of the overall variation in the experimental data. The eigenvalues of PC1 and PC2 were higher than 1.78. The variables (< 90˚) apart on the loading plot are positively correlated and strongly correlated. Accordingly, serotonin, MDA, dopamine, immobility time (FST), anxiety index (EPM), number of trials (MWM), latency in outer edge (OFT), Zn content, GAP-43 immunoexpression, and neurofilament disposition variables are grouped together and highly correlated with PC1. Moreover, the TAC was correlated with PC2 and was highly negatively correlated with PC1variables.

## Discussion

In the current work, Sprague Dawley rats were utilized as a model for investigating the BV role in rescuing the neurobehavioral dysfunction resulting from ZNP exposure as they show brain and behavior changes similar to those seen in various neurobehavioral diseases in humans (Chong et al. 2019; Flanigan et al. 2022; Ji et al. 2020). Besides, Sprague Dawley rats have more advantageous for neurotoxicity studies due to their ease of handling and calmness which facilitate conducting neurobehavioral tests and consequently have been used in many recent studies as a model for assessing the neuroprotective therapeutics (Le Roux et al. 2021; van Onselen et al. 2020). Moreover, male rats were used to avoid the variable nature of female data caused by hormonal variations associated with the female reproductive cycle (Chong et al. 2019; Yoest et al. 2018).

Initially, the tested ZNPs have an average particle size of 30 nm as revealed by SEM and TEM microscopical analysis. Regarding the size, several earlier reports have confirmed that this size of ZNPs was small enough for the NPs to be assimilated very easily by cells in the rat which had several folds of magnitude bigger size than the ZNPs (Shang et al. 2014; Tang et al. 2019). Moreover, various studies confirmed the toxicity of ZNPs at comparable sizes in rats (Dkhil et al. 2020; Xiaoli et al. 2017).

The change in body weight is one of the most widely used parameters in toxicological assessments to indicate the toxic effects of a substance on the health status of animals (Wang et al. 2019a). Thus, in most medical and environmental toxicology research on the effects of certain substances on the body, animal body mass and organ mass have been frequently measured (Abo-El-Sooud et al. 2023; Fadil et al. 2023). In the current study, ZNPs-exposed rats showed a significant reduction in daily weight gain compared to their respective control. Several reasons could be responsible for ZNPs-associated weight retardation, like its adverse effect on energy metabolism (Yan et al. 2012) and copper and iron metabolism (Yanagisawa et al. 2009). Besides, Yan et al. (2012) verified that the amount of ZNPs remaining in the intestine of rats following repeated oral exposure could disrupt the intestinal brush border and gut flora because of its antibacterial activity. On the other hand, the BV-treated groups exhibited an obvious restoration of their body weight gain. Similarly, Han et al. (2006) stated that stinging or subcutaneous injection of BV increased the body weight of piglets insignificantly. Administration of BV improved the body weight, which may be due to improved glycemic control (Mousavi et al. 2012).

Both the OFT and EPM tests have been used extensively to predict anxiety behaviors in murine models (Carola et al. 2002). Based on the findings of both OFT and EPM tests in the present study, ZNPS exhibited an obvious anxiogenic effect. In this respect, de Souza et al. (2018a) speculated that the anxiety caused by exposure to ZNPs may be because these NPs are small enough to bioaccumulate in the central nervous system and that they disrupt the normal functioning of neurological circuits involved in anxiety. Numerous neurotransmitters are implicated in the neurobiological controlling of anxiety (Mendes et al. 2017; Pałasz et al. 2021). Serotonin and dopamine signaling is shown to be positively involved in the pathogenesis of anxiety (Akimova et al. 2009; Jia & Pittman 2015; Murphy et al. 2013). In the current experiment, ZNPs-exposed rats displayed a significant increase in serotonin and dopamine brain content. ZNPs may have affected the anxiogenic systems, which are composed of excitatory amino acids, cholecystokinin, noradrenaline, and serotonin (Pałasz et al. 2021). From another perspective, ZNPs can generate ROS and damage membranes (Reddy et al. 2007), which may explain the anxiogenic-like effects observed in animals after ZNP exposure. The ZNP-induced oxidative stress was apparent in the brain tissues of ZNPs-exposed rats by decreased TAC content and increased MDA levels.

On the contrary, the BV-treated groups showed noticeably reduced anxiety in both OFT and EPM tests. Since there is a significant negative correlation between anxiety and the neurotransmitter levels in this study, this may be related to the beneficial effect of BV on these levels. Of note, the administration of BV in the current study significantly reduced the increased levels of brain neurotransmitters in BV + ZNPs-treated rat pups. Correspondingly, BV decreased rotenone-induced changes in neurochemistry in mouse brain models (Khalil et al. 2015b). Melittin, a strongly basic 26-amino-acid polypeptide that makes up 40% to 60% of BV, is thought to be responsible for this result because of its strong surface activity on cell lipids, which facilitates the translocation of BV along neural pathways and has a significant effect on neurotransmitter release (Chen and Lariviere 2010). Additionally, it has been found that BV protected tyrosine hydroxylase-containing neurons in the *substantia nigra pars compacta* of MPTP-treated mice (Kim et al. 2011). The peptide apamin and other neurotransmitters found in BV may also have a role in the drug’s effects (Chen and Lariviere 2010). For instance, cell culture studies showed that melittin inhibited the binding of D2 receptors with an antagonist and reduced dopamine uptake by acting directly on the dopamine transporter (Keith et al. 2011, 2012). In HEK-293 cells, Keith et al. (2011) showed that melittin, or an endogenous molecule triggered by melittin, directly interacted with the dopamine transporter.

The FST has proven to accurately predict depression in laboratory animals (Slattery and Cryan 2012). Herein, the findings of the FST revealed that rats in the ZNPs group remained motionless for longer, implying a depressant-like behavioral effect. The earlier findings are contrary to Xie et al. (2012a) study FST results in which male Swiss mice intraperitoneally injected with 5.6 mg/kg ZNPs were immobile for a shorter time, reflecting an antidepressant-like behavioral effect. According to Krishnan et al. (2007) and Wallace et al. (2009), anxiety can frequently occur as part of a depressive syndrome; however, this is not the case for all patients and certainly not for all animal models, as found in our work. That would explain the anxiogenic impact of ZNPs in animals. On the contrary, the BV-treated rats showed reduced depression. Comparably, Abd El-Wahab and Eita (2015) found that volunteers who received live bee sting acupuncture had considerably lower depression. Moreover, Cho et al. (2012) demonstrated that acupuncture alleviated depression accompanying Parkinson’s disease. The authors suggested that the drop in adrenocorticotropin and corticosterone plasma hormone levels associated with acupuncture could influence depression-related behavior. Besides, apamin, a naturally occurring peptide found in BV, is well-known for its capacity to block a type of ion channel that allows for a high-speed and selective passage of potassium ions out of nerves (Mourre et al. 1997). Blocking these channels in the brain leads nerves to become hyper excitable, resulting in better learning and implications for depression and dementia treatment (Alvarez-Fischer et al. 2013).

Notably, in the present study, ZNPs-exposed rats displayed obvious memory and learning impairments. Oxidative stress and mitochondrial dysfunction contribute to spatial learning and memory dysfunction (Mehdizadeh et al. 2017; Sharif et al. 2016). In the current study, the ZNPs induced oxidative stress in brain tissue was evidenced by the reduced TAC. Moreover, mitochondrial dysfunction has been proposed as the main underlying mechanism of ZNPs cytotoxicity (Patrón-Romero et al. 2022). Once ZNPs are internalized in the cell, they are distributed in all the organelles, particularly in the mitochondria (Wang et al. 2019b). Xia et al. (2008) verified that ZNPs interrupted cellular Zn homeostasis, resulting in mitochondrial damage and cell death. Hence, a potential explanation for the memory and learning deficits in ZNPs-exposed rats might be oxidative stress and mitochondrial dysfunctions. Another important perspective is that in the study of Slekiene et al. (2022), the incubation of amyloid-beta (Aβ) proteins with ZNPs caused morphological alterations and encouraged their fibrillation and aggregation. Aβ deposition has been reported to cause harmful events, including neuronal dysfunction, neurofibrillary tangle formation, and impaired memory (Haass and Selkoe 2007; Shankar et al. 2008)

On the contrary, the BV-treated rats displayed a significant enhancement of memory and spatial learning. This could be related to the improved oxidative status in the brain tissues associated with BV injection. BV’s antioxidant properties have already been established (Sobral et al. 2016). Our previous research with rat pups demonstrated that BV could be a preventative measure and a treatment against PPA-induced oxidative stress in the brain (Khalil et al. 2015a). Correspondingly, subcutaneous injection of rabbits with increasing doses of BV (0.1-0.3 mg/kg b.wt) for 20 weeks considerably augmented the GST activity and increased GSH level but reduced MDA (El-Hanoun et al. 2020). Mohamed et al. (2019) also verified the BV antioxidant protective effects in rats against acetylsalicylic acid-induced gastric ulceration. Likewise, the potent BV-antioxidant activity has been demonstrated in Freund’s Complete Adjuvant-induced arthritis model in rats in the study of Kocyigit et al. (2019). On the other hand, previous research has shown that BV protected the lumbar spinal cord of mice via controlling mitochondrial structure and cristae morphology (Yang et al. 2010). Also, the normalization of the behavior mentioned above in the present study may be attributable to the diverse biological components of BV. For instance, it has been shown that the principal ingredient of BV, melittin, and the enzyme phospholipase A2 (PLA2) effectively scavenge free radicals (Kim et al. 2002; Park et al. 2007). Besides, lipopolysaccharides-stimulated mice treated intraperitoneally with PLA2 (0.2 and 2 mg/kg) showed decreased expression of the amyloidogenic and inflammatory amyloid precursor protein and reduced memory impairment (Lee et al. 2008). Of note, in the studies of Ye et al. (2016) and Baek et al. (2018), BV PLA2 treatment dramatically reduced Aβ protein accumulation in the brains of mice and ameliorated cognitive deficits. The activation of the Aβ processing enzymes, such as secretases, is controlled by neuronal activity and facilitates memory and learning (Ma et al. 2007). Interestingly, Ham et al. (2019) demonstrated that BV PLA2 could repair memory through inhibiting the Aβ accumulation and regulating β-secretase activity.

The Zn accumulation following nano-Zn exposure has been proposed as a possible mechanism for organ damage. It has been responsible for oxidative stress, DNA damage, and apoptosis in the affected organ (Sharma et al. 2012). The current study recorded significant concentrations of Zn in the brain tissues of ZNPs-exposed rats. Comparably, ZNPs could reach the brain after oral, inhalation, and intraperitoneal administration in animals (Kao et al. 2012; Lee et al. 2012; Tian et al. 2015) and impair the memory and spatial learning of rats by changing the synaptic plasticity and interacting with brain proteins (Shim et al. 2014). Besides, several studies showed that NPs (including ZNPs) in circulation could change blood–brain barrier (BBB) integrity and permeability via the following mechanisms: endothelial cell oxidative stress (Setyawati et al. 2013), NPs-induced endothelial cell leakiness (Setyawati et al. 2015), and inflammatory pathway (Giovanni et al. 2015). Then, NPs and/or pro-inflammatory mediators could penetrate the brain and induce harmful consequences (Tian et al. 2015). It is important to mention that the dissolved zinc ion (Zn^2+^) in the target organs or the cell culture medium is mostly responsible for the toxicities of ZNPs (Song et al. 2010). ZNPs may cause membrane damage by generating ROS after interacting directly with the outer membrane surface (Patel et al. 2016). Instead, the brain of the ZNPs-exposed group but treated with BV had significantly lower Zn content than those exposed to ZNPs. In this regard, in a recent study by Linville et al. (2021), melittin supports the BBB transient, opening a novel technology for the delivery of therapeutics into the brain. Also, an important feature of apamin is its capacity to cross the blood–brain barrier and reach the central nervous system (Palma 2013). Interestingly, in the recent study of Abu-Zeid et al. (2021), treatment with BV from *Apis mellifera lamarckii* retained a promising in vivo protective ability against methyl mercury-induced BBB dysfunction. Thus, BV bioactive’s capacity to penetrate BBB while maintaining its function could be responsible for reducing Zn harmful accumulation in brain tissues.

GAP-43, a protein widely distributed in neurons, plays a crucial role in memory and learning (Benowitz et al. 1990; Holahan et al. 2007). According to Holahan et al. (2007), modest overexpression of the phosphorylatable plasticity-related protein, GAP-43, can improve memory, whereas excessive overexpression can result in a "neuroplasticity load" with hypertrophic and degenerative processes resulting in memory impairment. This was apparent in the current study, the ZNPs-exposed rats showed a significant over-immunoexpression of GAP-43 while the BV-treated great showed moderately increased GAP-43 immunoexpression. Besides, as recorded in the existing behavioral findings, memory impairment was obvious in ZNPs-exposed rats, but memory enhancement was evident in the BV -treated ones. Moreover, Rekart et al. (2004) detected high GAP-43 protein levels in Alzheimer’s patients brains. The earlier study suggested that GAP-43 overexpression may be linked to memory problems, though whether this was a cause or a consequence remains unclear.

Neurofilament proteins are key neuron-specific components that sustain structural integrity and are vulnerable to neurodegeneration and neuronal damage in various neurologic disorders. Throughout normal brain growth, maturation, and aging, neurons release low quantities of NfPs into the extracellular space, which reach the cerebrospinal fluid and blood. Neurodegeneration and damage increase NfP levels (Yuan and Nixon 2021). Herein, a considerable increase in the immunoexpression of neurofilament was recorded in the brain sections of ZNPs-exposed rats, reflecting tissue injury. Similarly, Liu et al. (2020) reported that ZNPs can damage nerve fibres, degrade neuronal structure, shorten neuritis, and diminish nerve nodes by injuring tubulin-β, tubulin-α, and NF-H. This disrupts neural network structure formation and nerve cell-neural network communication. Notably, a strong positive correlation exists between neurofilament and GAP-43 in the current study. In this regard, Chung et al. (2020) demonstrated that neuronal and glial damage releases neurotrophic factors and cytokines, which activate growth-associated proteins like GAP-43 to protect and regenerate neurons. On the contrary, BV treatment significantly alleviated the ZNPs-induced overexpression of neurofilament. This could reflect the BV-associated repair of the neuron injury, possibly related to its antioxidant activity.

Despite BV’s potential therapeutic value in mitigating the neurobehavioral aberrations and neurotoxic effects of ZNPs, its safety profile is an essential consideration, as immune responses to BV therapy can range from mild skin reactions that clear up after a few days to severe, potentially fatal reactions like anaphylaxis (Park et al. 2013). Some trials showed no adverse events or systemic reactions associated with BV therapy (Nagai et al. 2004), while others observed minor adverse events but no major systemic reactions (Castro et al. 2005). Park et al. (2015) recently proposed that patient education materials on the safety and efficacy of BV should be made available to increase the likelihood that BV would be used successfully as a medication. Practitioners should also be aware of the adverse effects of BV, establish clinical recommendations to reduce adverse events, and design and apply strict criteria for monitoring adverse events.

## Conclusion

The current study’s findings indicate that BV could be play a significant alleviative role against neurobehavioral problems and neurotoxic impact caused by ZNPs. This beneficial role could be mediated by adjusting neurotransmitter levels, maintaining brain zinc concentration, increasing antioxidant activity, and decreasing GAP-43 and neurofilament expression in brain tissue. Hence, human studies are desperately needed to establish if BV could be utilized as a beneficial strategy in persons likely to be exposed to ZNPs, such as those working in the manufacturing industry. More research is required to understand further the other potential mechanism by which BV provides neuroprotection.

## Data Availability

All data generated or analyzed during this study are included in this published article.
